# 751 Incidence of Pavement Burns During a Worldwide Pandemic and Limited Outreach Efforts

**DOI:** 10.1093/jbcr/irac012.304

**Published:** 2022-03-23

**Authors:** Yasmin Conaway, Maria Ortega-Polanco

**Affiliations:** University Medical Center of Southern Nevada, Las Vegas, Nevada; University Medical Center of Southern Nevada, Las Vegas, Nevada

## Abstract

**Introduction:**

Areas of the United States with desert climates and consistent ambient temperatures greater than 100 degrees Fahrenheit four to five months out of the year have a significant influx of pavement burn inpatient admissions during the summer season. These burns are caused when skin meets elevated pavement temperatures, even if the contact is just for a few seconds. We investigated retrospective data and the impact of the decreased outreach efforts during the pandemic on pavement burn admissions.

In 2018, due to an identified increase in pavement burn admissions in the community, the burn center created and implemented a pavement burn campaign to reduce pavement burn admission by raising awareness and providing education in the community. The campaign included specific messaging posted on billboards, public service announcements via news outlets and social media, printed flyers distributed during various community safety events, and the distribution of summer footwear between the months of April and September. The efforts proved fruitful in reducing rates. Due to the worldwide pandemic, prevention efforts were greatly decreased in 2020.

**Methods:**

A 3-year retrospective data review of acute admissions to a 16-bed burn unit was conducted. Data was abstracted from the burn center’s registry, looking at rates of pavement burns within the months of April to September. Each of the three year’s inpatient pavement burn admission totals were compared.

**Results:**

Our analysis showed a need for burn prevention strategies in 2018, leading to implementation of the pavement burn campaign. There was a 58% decrease in admission rates in 2019 compared to 2018.

In March of 2020, the state ordered businesses to close; additional closures and restrictions continued over the summer months causing all prevention efforts to cease. In 2021, there was a 60% increase in pavement burn admissions compared to previous two years. There is a correlation between halted pavement outreach and the increase in pavement burn admissions.

**Conclusions:**

The lack of burn outreach during the pandemic lead to a remarkable increase in pavement burn admissions in 2021, reinforcing the need for continued outreach in the hot summer months. Additional research is needed to identify specific populations admitted for pavement burns to identify needs of the community. Patient demographics such as age and geographical location can aid in targeting outreach strategies for the identified populations. Additional research with other burn centers may aid in constructing innovative outreach strategies moving forward.

